# Distal Allograft Pancreatectomy After Pancreas Transplantation: A Case Report and Review of Literature

**DOI:** 10.1155/crit/6655739

**Published:** 2026-05-04

**Authors:** Eriselda Keshi, Brigitta Globke, Charlie Alexander Hamm, Andreas Kahl, Johann Pratschke, Robert Öllinger, Nathanael Raschzok

**Affiliations:** ^1^ Department of Surgery, Campus Charité Mitte, Campus Virchow-Klinikum, Charité–Universitätsmedizin Berlin, Berlin, Germany, charite.de; ^2^ Berlin Institute of Health (BIH), Berlin, Germany, bihealth.org; ^3^ Clinic for Radiology, Charité–Universitätsmedizin Berlin, Berlin, Germany, charite.de; ^4^ Department of Nephrology, Campus Charité, Benjamin Franklin, Charité–Universitätsmedizin Berlin, Berlin, Germany, charite.de; ^5^ Cluster of Excellence Matters of Activity, Image Space Material Funded by the German Research Foundation (DFG) Under Germany′s Excellence Strategy—EXC 2025—390648296, Berlin, Germany

## Abstract

Paraneoplastic lesions such as intraductal papillary mucinous neoplasms (IPMNs) are uncommonly observed in the graft of recipients of pancreas transplants, despite the well‐established association between immunosuppression and malignancy development. Typically, allograft pancreatectomy is performed due to complications such as acute rejection or vascular thrombosis. We report a rare case of a patient who developed histologically confirmed IPMN occurring 8 years after pancreas transplantation. As the detection of paraneoplastic lesions increases with advancing imaging techniques and pancreas allograft survival continues to improve, these findings may gain greater clinical relevance. This case underscores important considerations for transplant surgeons, particularly regarding posttransplant surveillance strategies, management approaches, and long‐term follow‐up.

## 1. Introduction

Vascularized pancreas transplantation, first successfully performed in 1966, offers complete beta cell replacement, restoring natural insulin regulation, and eliminating the need for glucose monitoring or insulin injections in diabetes patients [1]. Simultaneous pancreas–kidney transplantation (SPK) is the treatment of choice for patients suffering from Type 1 diabetes mellitus with end‐stage renal disease and is now being commonly performed [1]. Advancements in immunosuppressive therapy, critical care, and surgical experience have improved the longevity of pancreatic allografts. However, despite lifelong immunosuppression and its known oncogenic risks, reports of preneoplastic or neoplastic lesions in pancreatic allografts remain rare. We present a case of a patient diagnosed with an intraductal papillary mucinous neoplasm (IPMN) during routine posttransplant surveillance. Rapid lesion growth prompted a transplant distal pancreatectomy. This case is unique in that it describes a rapidly progressive IPMN with high‐grade epithelial dysplasia arising in a functioning pancreas allograft, successfully managed with distal pancreatectomy while preserving graft function and insulin independence, an outcome rarely reported in the literature.

## 2. Case Report

Written informed consent was obtained from the patient for the publication of this case report and any accompanying images. The patient is a 65‐year‐old male with a complex medical history, including longstanding Type 1 diabetes mellitus (diagnosed in 1983), complicated by diabetic nephropathy and progression to end‐stage renal disease, requiring hemodialysis from 2011 to 2016. He was treated at the Department of Nephrology and the Department of Surgery, Charité–Universitätsmedizin Berlin. His chronic kidney disease (CKD) was associated with renal anemia, hypertension, metabolic acidosis, secondary hyperparathyroidism, and renal osteopathy. Cardiovascular comorbidities include arterial hypertension, a 30 pack‐year smoking history, and coronary artery disease, status postpercutaneous coronary intervention with drug‐eluting stent placement in the left anterior descending artery. Gastrointestinal assessment revealed diabetic gastroparesis and significant cardia insufficiency, confirmed by upper endoscopy in May 2017. He also suffers from advanced diabetic retinopathy with profound visual impairment. In January 2017, he underwent bilateral nephrectomy due to renal cell carcinoma.

The patient received a SPKT in November 2016. The donor was a 43‐year‐old female who died from subarachnoid hemorrhage secondary to a ruptured cerebral aneurysm, with no other known comorbidities. The organ donation was performed in accordance with national regulations and ethical standards. Consent for organ donation was obtained through the appropriate legal framework. The early posttransplant course was complicated by episodes of acute rejection in December 2016 and February 2017, both treated with high‐dose corticosteroids. He also experienced opportunistic infections, including cytomegalovirus (CMV), *Candida* esophagitis, and *Clostridium difficile* colitis. In May 2022, he developed an episode of acute kidney allograft injury (KDIGO Stage I), characterized by a transient rise in serum creatinine from 2.0 to 3.6 mg/dL. His maintenance immunosuppression regimen includes tacrolimus, mycophenolate mofetil, and methylprednisolone.

**Figure 1:**
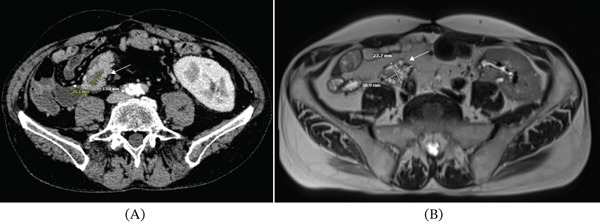
Follow‐up of the cystic pancreatic lesion using (A) contrast‐enhanced CT in March 2024 and (B) T2‐weighted MRI in November 2024, showing a substantial increase in size within a short period of time.

Eight years after transplantation, routine follow‐up CT imaging revealed a lesion of uncertain dignity in the tail region of the pancreas transplant (Figure 1). After interdisciplinary discussion, an open surgical exploration was indicated, with the possibility of a distal pancreatic resection or total transplant pancreatectomy, pending histological confirmation. Surgery was performed on April 16, 2024. Intraoperative frozen section analysis revealed no evidence of malignancy but raised suspicion for an IPMN, without definitive features of high‐grade dysplasia or invasive carcinoma. Given the absence of confirmed malignancy, the potential risk of graft loss associated with immediate resection, and the patient′s stable clinical condition, the decision was made in an interdisciplinary setting to defer resection pending final histopathological evaluation. Apart from a transient increase in inflammatory markers, empirically treated with piperacillin/tazobactam, the postoperative course was uneventful. Extensive diagnostic workup revealed no infectious focus. Both pancreatic and renal graft function remained stable, with normal perfusion demonstrated by Doppler ultrasound. Immunosuppression was continued with regular monitoring of trough levels. Final histopathology confirmed IPMN with high‐grade epithelial dysplasia.

Follow‐up imaging in June and August 2024 showed a stable lesion. However, magnetic resonance imaging (MRI) in November 2024 revealed multiple progressive cystic lesions in the pancreatic tail (up to 30.9 × 23.7 mm; previously 20.1 × 13.4 mm) and progressive dilation of the pancreatic duct (up to 6 mm; previously 4 mm). The case was again reviewed in an interdisciplinary tumor board, and an indication was established for either a distal or total allograft pancreatectomy.

**Figure 2:**
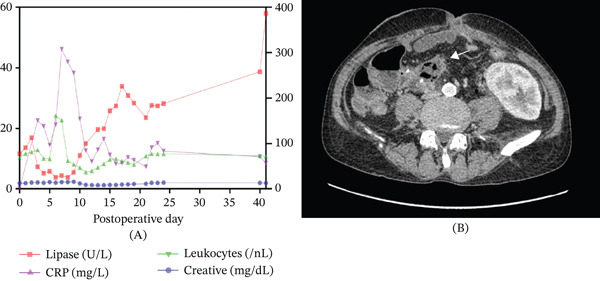
(A) Postoperative trends in serum lipase, CRP, leukocyte count, and creatinine levels showing transient inflammatory response and isolated lipasemia. Reference ranges: lipase (13–60 U/L), CRP (< 5 mg/L), leukocytes (4–10 × 10^9^/L), creatinine (0.7–1.3 mg/dL). (B) Axial contrast‐enhanced CT image demonstrating a large pelvic fluid collection with air–fluid level suggestive of abscess formation.

A distal allograft pancreatectomy was performed on December 15, 2024. Following adhesiolysis of omental remnants and small bowel loops, the pancreatic tail was fully mobilized. Intraoperative ultrasound guided the resection just beyond the fibrotic‐appearing lesion. The splenic artery and vein were isolated and ligated, and resection was completed using an EndoGIA stapler (Figure S1). Frozen section confirmed high‐grade dysplasia and free resection margins. The early postoperative course was uncomplicated, with stable glucose levels (no insulin therapy required) and preserved renal function. On postoperative Day 6, the patient presented with abdominal pain, fever, nausea, vomiting, and elevated inflammatory markers (leukocytosis and elevated CRP, Figure 2A). Emergency CT revealed a fluid collection extending pararectally on the left and cranially toward the bladder, measuring up to 8.5 × 10.5 cm, with a prominent air–fluid level but no peripheral contrast enhancement (Figure 2B). CT‐guided drainage was performed, with continuous irrigation at 50 mL/h. Initially, purulent fluid was drained, followed by feculent output on the next day.

A relaparotomy was performed on December 22, 2024, via the prior midline incision. The CT drain was found within a feculent, putrid cavity and removed. The collection extended to the pancreatic stump, which appeared intact with no evidence of pancreatitis. Exploration identified a perforated sigmoid diverticulum as the source of peritonitis. The sigmoid colon was inflamed and thickened, especially the epiploic appendages. After debridement, tangential sigmoid resection was performed using an Echelon‐powered stapler with three reloads. Due to serosal fragility, the staple line was reinforced with interrupted 3‐0 Vicryl sutures. The abdominal cavity was irrigated with 4–5 L of saline. Given the feculent peritonitis, temporary open abdomen management with abdominal dressing was initiated. On postoperative Day 1, the abdomen was re‐evaluated. Turbid but nonfeculent fluid was noted; the upper abdomen remained clean. The sigmoid staple line was intact and reinforced with Vicryl. Air leak testing via peranal Foley catheter confirmed an intact anastomosis. The pancreatic staple line appeared clean, covered with TachoSil, and without evidence of leakage or necrosis. Lavage with 4–5 L of saline was performed again. On December 27, 2024, a third‐look operation was conducted. After thorough lavage, two easy‐flow drains were placed via the right lower abdomen—one directed toward the pelvis and the other paracolic on the right. Fascial closure was achieved using two PDS loop sutures.

The patient required ICU management for 9 days and was transferred to the general ward on December 31, 2024. Recovery was uneventful. Antimicrobial therapy included meropenem, caspofungin, and linezolid, which were discontinued on January 3, 2025. During the septic episode and subsequent surgical interventions, immunosuppressive therapy was adjusted. Mycophenolate mofetil was temporarily withheld, and tacrolimus dosing was reduced with close monitoring of trough levels. Corticosteroids were continued to maintain baseline immunosuppression and prevent acute rejection. On January 10, 2025, due to laboratory‐confirmed lipasemia without clinical or functional impairment of the pancreatic graft, a follow‐up CT scan was performed. Imaging revealed morphologically regressing transplant pancreatitis following partial pancreatectomy, with no peripancreatic collections or evidence of anastomotic insufficiency at the duodenojejunostomy. Given his stable condition, the patient was discharged the same day.

Final histopathological analysis of the resected pancreatic tail confirmed an extensive IPMN with high‐grade intraepithelial neoplasia, characterized by complex papillary architecture, epithelial stratification, and marked cytological atypia. The lesion measured up to 40 mm. Resection margins were negative, with a minimal clearance of 7 mm from the stapled transection line. Chronic fibrosing and focally acute necrotizing pancreatitis with interstitial fibrosis and loss of exocrine tissue was also present. All five peripancreatic lymph nodes were free of tumor (0/5). No invasive carcinoma was identified on serial sectioning and immunohistochemical analysis. Due to the presence of high‐grade dysplasia, the case was reported to the cancer registry.

## 3. Discussion

Although there are a lot of developments on the management of IPMNs, there are currently not enough reports of pancreas allograft IPMNs and their surgical management, and clinical guidance for their management is limited. The present case adds to a growing, yet still sparse, body of literature, highlighting the complexity of diagnosing and treating cystic pancreatic neoplasms in the posttransplant setting.

In a 2018 case series, Al‐Qaoud et al. reported that 18% of 1185 pancreas transplant recipients were found to have IPMNs. Although most cases involved small branch duct IPMNs that were managed conservatively, one developed concerning features, including ductal dilation and cyst growth, so transplant pancreatectomy was performed [2]. Similarly, our patient developed progressive cystic changes and main duct dilation, ultimately leading to an indication for surgery.

Although the application of the Fukuoka guidelines in pancreas allografts may be limited by the lack of endoscopic ultrasound (EUS) in certain anatomical configurations, cross‐sectional imaging in our case clearly demonstrated worrisome features, including lesion size greater than 30 mm, main duct dilation, and interval growth. These findings appropriately guided the indication for surgical resection [2, 3]. In our patient, EUS was not technically feasible due to the specific transplant anatomy, with the pancreas allograft located in the right iliac fossa and enteric drainage via a duodenojejunostomy, limiting adequate endoscopic access to the graft. Therefore, diagnosis and surveillance relied on cross‐sectional imaging. These limitations, along with concerns that long‐term immunosuppression may accelerate cyst growth or increase the risk of malignant transformation, underscore the need for modified algorithms for surveillance in transplant recipients [2]. MRI with MRCP may offer superior sensitivity over CT for ductal pathology and should be considered the modality of choice where feasible [4].

The role of immunosuppression in the natural history of IPMNs remains poorly defined. Although studies have shown that the risk of developing a malignancy from a benign pancreatic lesion is not higher for transplanted patients, close follow‐up is indicated [5–7] as the chronic use of immunosuppressants theoretically impairs immune surveillance and could accelerate neoplastic progression. Agents such as calcineurin inhibitors have been implicated in promoting tumorigenesis through mechanisms involving TGF‐ß signalling and reduced DNA repair [8]. Although causality cannot be established for sure in our case, the presence of high‐grade dysplasia in a relatively short time frame supports the need for close surveillance in these patients.

The first report of a surgically resected IPMN in a transplanted pancreas was published by Serrano et al. [9]. They described the case of a 58‐year‐old man with Type 1 diabetes and a history of pancreas transplant in 2001 who was found to have a cystic lesion in his graft pancreas, later confirmed as an IPMN, during evaluation for recurrent hematuria 15 years after transplantation. He underwent a distal pancreatectomy with enteric conversion, and pathology revealed a low‐ to intermediate‐grade IPMN; postoperatively, he recovered well with stable graft function and no further hematuria. Indeed, surgical decision‐making in the transplant context poses unique challenges. In the native pancreas, resection is guided by the balance between oncologic necessity and surgical risk. However, in transplant patients, this decision is further complicated by the need to preserve endocrine function and maintain graft viability. Similar to Serrano et al., in our case a distal pancreatectomy allowed for complete resection, whereas sufficient pancreatic tissue was preserved to maintain insulin independence. Although they have described successful distal pancreatectomy for IPMN in a pancreas allograft, those cases involved low‐ to intermediate‐grade lesions. In contrast, our case is distinguished by rapidly progressive disease with high‐grade dysplasia, requiring timely surgical intervention while preserving graft function.

Interestingly, our patient developed a postoperative complication, which was the perforated sigma diverticulitis. Although the complication represents a separate pathological entity, it is plausible that surgical factors such as extensive adhesiolysis, combined with immunosuppression‐related tissue fragility, contributed to its occurrence. This underscores the multifactorial nature of postoperative complications in transplant recipients. Our patient also developed one graft‐related complication, which was the pancreatitis of the remaining pancreas allograft, detected through increasing blood lipase levels. However, the condition was clinically silent, uncomplicated, and showed spontaneous regression on subsequent follow‐up imaging. At the most recent follow‐up in September 2025, the patient was clinically stable, with preserved pancreatic and renal allograft function and no radiological evidence of disease progression.

In conclusion, this case highlights several important clinical lessons. First, IPMNs can arise in pancreas allografts and may demonstrate rapid progression to high‐grade dysplasia, underscoring the need for vigilant long‐term surveillance in transplant recipients. Second, cross‐sectional imaging plays a central role in diagnosis and monitoring, particularly when EUS is not feasible due to altered anatomy. Third, graft‐preserving distal pancreatectomy can be a safe and effective surgical option in selected patients, allowing oncologic control while maintaining endocrine function. Given the potential for multifocal disease and malignant transformation, structured lifelong imaging surveillance of the remaining allograft appears warranted. In our practice this includes regular follow‐up using MRI at defined intervals (e.g., every 6–12 months depending on imaging findings), to enable early detection of recurrence or progression. Further studies are needed to better define optimal management strategies in this rare clinical setting.

## Funding

Open access funding was enabled and organized by Projekt DEAL.

## Ethics Statement

Approval was not required.

## Consent

Informed written consent was given by the patient.

## Conflicts of Interest

The authors declare no conflicts of interest.

## Supporting information


**Supporting Information** Additional supporting information can be found online in the Supporting Information section. Figure S1 Schematic illustration of the pancreas allograft anatomy. The graft is located in the right iliac fossa with arterial inflow via a Y‐graft anastomosed to the external iliac artery and systemic venous drainage via the portal vein. Exocrine drainage is established through a duodenojejunostomy. The intraductal papillary mucinous neoplasm (IPMN) is located in the pancreatic tail. The dashed line indicatesindicated the level of distal pancreatectomy.

## Data Availability

Data sharing is not applicable to this article as no datasets were generated or analyzed during the current study.
