# Supplementary figures and images for: Distal Allograft Pancreatectomy After Pancreas Transplantation: A Case Report and Review of Literature
Source: Case Rep Transplant. 2026 May 4;2026:6655739. doi: 10.1155/crit/6655739 (PMC13137291; doi:10.1155/crit/6655739)

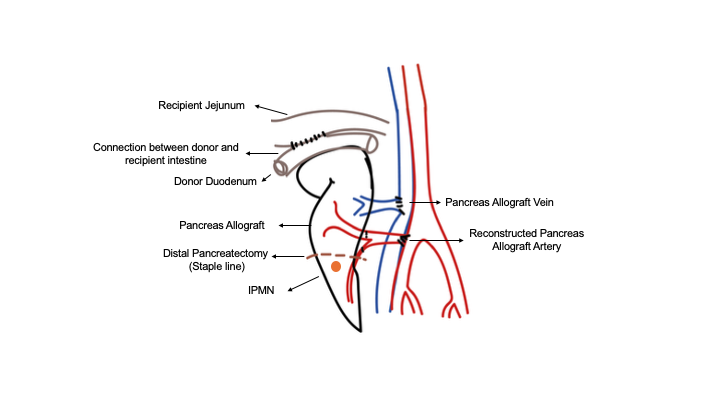

Supplement: Supplementary file 1 — Supporting Information Additional supporting information can be found online in the Supporting Information section. Figure S1 Schematic illustration of the pancreas allograft anatomy. The graft is located in the right iliac fossa with arterial inflow via a Y‐graft anastomosed to the external iliac artery and systemic venous drainage via the portal vein. Exocrine drainage is established through a duodenojejunostomy. The intraductal papillary mucinous neoplasm (IPMN) is located in the pancreatic tail. The dashed line indicatesindicated the level of distal pancreatectomy. [file CRIT-2026-6655739-s001.png]
